# Application of Plant-Based Hydrocolloids on the Textural Profile of Vegan Gummies Supplemented with Turmeric and Black Pepper

**DOI:** 10.1155/2024/7127635

**Published:** 2024-04-22

**Authors:** Santoshi Rawat, Sweta Rai, Sabbu Sangeeta, Anil Kumar, Preethi Ramachandran, Satish Kumar Sharma, Shiv Kumar Dubey, Arun Prakash, Riya Joshi

**Affiliations:** ^1^Department of Food Science and Technology, College of Agriculture, Govind Ballabh Pant University of Agriculture and Technology, Pantnagar, Uttarakhand, India; ^2^Department of Biochemistry, College of CBSH, Govind Ballabh Pant University of Agriculture and Technology, Pantnagar, Uttarakhand, India

## Abstract

Gummies belong to a confectionery category characterized by a hydrocolloid, acting as a stabilizer, forming a network to retain a high-moisture sugar syrup, and hydrocolloids play a key role in shaping the visual appeal, flavour release, and texture of the gel network. This study investigates the potential substitution of gelatin in gummies with plant-based hydrocolloids like agar-agar and guar gum. It is also aimed at optimizing the level of functional ingredients like curcumin and piperine in standardized gummies through incorporation of turmeric and black pepper, respectively. These plant-based gelling agents mimic gelatin's chewable, firm, and elastic texture, catering to broader consumption and suitability for versatile use. Consumer interest in healthier diets has spurred the transition towards plant-based functional foods, leading to the replacement of gelatin gummies with plant-based alternatives. Agar-agar significantly influences gummy texture by contributing to firmness, elasticity, and stable gel formation, imparting essential strength and consistency. Guar gum, recognized as a plant-based hydrocolloid, enhances gummy texture, consistency, and moisture retention through thickening and stabilization. While agar-agar and guar gum individually fell short in achieving the desired textural attributes in the gummies, their combined use (1% agar-agar and 5.5% guar gum) yielded optimal chewiness (1,455.12 ± 1.75 N), gumminess (2251.11 ± 2.14 N), and high overall acceptability (8.96), resembling gelatin-based gummies. The optimized formulation included 40% sugar, 2% citric acid, 2% turmeric, and 0.6% black pepper. The developed vegan gummies contained 56.9 ± 0.09 mg/100 g total phenols, 37.27 ± 1.4% antioxidant capacity, 0.054 ± 0.0012% curcumin, and 0.02 ± 0.008% piperine. Consequently, the combined use of agar-agar and guar gum emerged as stable and effective gelling agents, offering an alternative to gelatin for creating turmeric and black pepper-infused gummies with desirable texture and functional attributes.

## 1. Introduction

The global confectionery sector is one of the fastest-growing sectors in the world with an estimated compound annual growth of 8.4% and represents a large group of high-calorie food which covers products ranging from candies, chocolate, cakes, bars, gummies, and other sweet foods [1]. The confectionery market includes sugar-boiled confectionery, hard-boiled candies, jelly candies, gummies, toffees, and other sugar-based candies [2]. Among these, gummy candy is a very popular confectionery product that represents approximately 50% of the candy market value [3]. Due to the unique and chewy nature of gummies, they are widely consumed by everyone regardless of age group. Gummies are a class of confections based on a hydrocolloid (sometimes called a stabilizer) that provides a network to hold relatively high-moisture sugar syrup. These hydrocolloids can influence appearance, flavour release, and textural attributes in the gel network [4]. Gummies consist of food products whose main ingredient is sugar, normally incorporated in the form of sucrose syrup or glucose syrup, combined with gelling agents, acids, aroma, and food colourants [5]. Gummy is consumed by a large and heterogeneous group of people [6]. Gummies are generally made up of gelatin, a protein derived from collagen, which gives a firm and unique elastic nature to gummy candy. This protein primarily comes from animal sources (cow, pig, etc.), which is generally not acceptable for consumption by vegan people [7]. The quest for alternatives to gelatin in gummies, particularly in achieving a chewy texture, has led to the exploration of plant-based hydrocolloids. Different studies highlight the potential of agar-agar and guar gum as effective gelling agents, offering a texture akin to gelatin in various food products [8]. These plant-derived hydrocolloids not only impart the desired chewiness but also address concerns regarding the ethical and religious considerations associated with animal-derived gelatin [9]. Therefore, leveraging agar-agar and guar gum presents a promising avenue for creating chewy textures in food formulations while catering to diverse dietary preferences. Hence, to obtain a chewy texture similar to gelatin, plant-based sources like agar-agar and guar gum can be the best alternative option as a gelling agent. It was seen in the last two years that many lives were affected by COVID-19, and this situation highlighted the increasing importance of functional ingredient-based immunity-boosting products [10]. Traditional spices and herbs usually contain functional ingredients that have the potential to provide health benefits. They improve health and supplement the basic daily requirements. They contain functional components that have received considerable attention due to their many recognized medicinal properties such as antidiabetic, antiseptic, antibacterial, antiasthmatic, antiulcer drug, antioxidant, anti-inflammatory, and wound healing properties [11]. However, due to their palatability, confectionery products are suitable carriers of functional ingredients to develop healthier products and effective daily supplements which can be consumed by everyone and are suitable for “anytime-everywhere” consumable products. Considering the popularity and convenience associated with confectionery products, they can be used as a vehicle for the incorporation of functional ingredients [12]. Considering the popularity and convenience associated with confectionery products, they can be used as a vehicle for the incorporation of turmeric and black pepper. Turmeric is a traditional medicine used in Ayurveda medicine for various diseases [13]. Turmeric and its active component curcumin have received considerable attention due to their many recognized medicinal properties. Curcumin is a potential therapeutic agent, but it has poor bioavailability due to poor absorption, rapid metabolism, and rapid systemic elimination. Similarly, black pepper (*Piper nigrum* L.) is also used in many food preparations and is known as the king of spices. It shows various medicinal properties and is found to be beneficial during fever or cold. To improve the bioavailability of curcumin, Shoba et al. studied and found that piperine from black pepper increases the bioavailability of curcumin by 2000%, when added 2 mg of piperine with 2 g of curcumin [14, 15]. While nutritionally supplemented gummies, such as vitamin C for immune support, biotin for hair health, and hyaluronic acid for skin benefits, have gained popularity in the market as nutraceutical gummies, there is a notable scarcity of gummies focused on immune-boosting functional ingredients like turmeric and black pepper, either individually or in combination [16]. Therefore, this study addresses the gap by developing vegan gummies supplemented with immune-boosting ingredients. It is aimed at assessing the impact of replacing gelatin with plant-based hydrocolloids on the texture of gummies infused with turmeric and black pepper. Additionally, the physicochemical properties of the final product will be analyzed.

## 2. Materials and Methods

### 2.1. Raw Materials and Chemicals

Food-grade agar-agar, guar gum, and citric acid were used. Agar-agar is an off-white powder with a gelling strength of 800+, which is a commercial product commonly used in the food industry. The guar gum had a viscosity ranging from 5500 cps to 6000 cps and a particle size of 200 mesh. These gelling agents' agar-agar and guar gum were stored at a cool (20 ± 2°C) and dry place (RH ≤ 60%). For citric acid, standard grade was used. Turmeric and black pepper powder were procured from a local market. Chemicals and reagents required during various types of sample analysis were AR grade and purchased from HiMedia Laboratories Pvt. Ltd. and SD Fine Chem. Ltd.

### 2.2. Moisture Content

The moisture content of raw ingredients and gummies was estimated by meshing the weighed sample and drying it to a constant weight in a hot air oven. The oven-dried samples were then cooled to room temperature in desiccators before weighing. The moisture content of the samples was then calculated and expressed as a percentage on a fresh weight basis [17].

### 2.3. Total Soluble Solids (TSS), pH, and Water Activity

TSS of the gummy sample was recorded by using the model Erma hand refractometer of the 60-90 range and expressed in terms of degree Brix. The sample was removed from the heating mantle after all ingredients had been cooked and allowed to cool at room temperature, and TSS was measured before setting the gummies. pH of gummies was determined by standard method [18]. pH of gummies was measured by a digital pH meter (HI96107, M/s Hanna Instruments, Navi Mumbai) at room temperature. For gummies, a 25 g sample was macerated in a pestle and mortar with added 25 mL distilled water and the pH of the suspension was measured. Water activity was estimated by a computer digital water activity meter (FA-st/1, GBX), where direct measurements were taken at room temperature.

### 2.4. Titratable Acidity (%)

The titratable acidity of gummies was determined by standard method [19]. To make a suspension, 10 g gummies were macerated in a pestle and mortar with 100 mL hot (60-70°C) distilled water. Measured 10 mL of aliquot of sample and acidity was estimated by titrating a known volume of sample against standard 0.1 N NaOH using phenolphthalein as an indicator. The titratable acidity was expressed as percent as per the given formula:

(1) $$\begin{array}{c} \frac{\text{Titre}\times \text{normality of alkali}\times \text{Vol}.\text{made up}\times \text{Eq}.\text{weight of dominating acid}}{\text{Weight of sample}\times \text{volume of aliquot}\times 1000\;}\times 100. \end{array}$$

### 2.5. Curcumin Content

Curcumin content was determined using the ethanol extraction method [20]. Standard curcumin solution was prepared by dissolving 25 mg of standard curcumin (AR grade, HiMedia Laboratories Pvt. Ltd., Mumbai), with ethanol (99% purity), into a 100 mL volumetric flask; 1 mL of this solution was transferred to another 100 mL volumetric flask and diluted to the meniscus with ethanol (99% purity). This standard solution contained 2.5 mg/litre of curcumin. About 1 g of ground, supplemented gummies was added to 30 mL ethanol (99% purity) in a round bottom flask separately and kept in a water bath for two and half hours. Curcumin extract was then cooled and filtered into a 100 mL volumetric flask. Pipette out 20 mL of the filtered extract into a 250 mL volumetric flask and dilute up to the meniscus. The absorbance of the sample was taken at 425 nm using a UV-visible spectrophotometer. The amount of curcumin was calculated by a standard curve using the following equation.

(2) $$\begin{array}{c} \text{Curcumin}\%=\frac{0.0025\times \text{absorbance}\times \text{Vol}.\text{made up}\times \text{dilution factor }}{0.42\times \text{sample weight }\left(\text{g}\right)\times 1000}\times 100. \end{array}$$

### 2.6. Piperine Content

The piperine content of black pepper powder and gummies was measured by using a standard method [21]. Accurately weigh 0.5 g gummy sample (ground in pestle and mortar) and black pepper powder, transfer to a 125 mL Erlenmeyer separately, and protect from light. Add 70 mL 1,2-dichloroethane (C_2_H_4_C_12_), reflux, and stir for 1 h. Extracted piperine was then cooled and filtered into a 100 mL volumetric flask. Then, pipette out 2 mL of this solution into another 100 mL volumetric flask, dilute up to the volume mark, and measure absorbance at a maximum of 345 nm using a UV spectrometer.

(3) $$\begin{array}{c} \text{Piperine}\%=\frac{A_{\text{s}}\times F\times V}{W_{\text{s}}\times 10^{6}}\times 100, \end{array}$$

where *A*_s_ is the absorbance of sample, *F* is the factor derived from piperine standard, *V* is the dilution volume (mL), and *W*_s_ is the sample weight (g).

### 2.7. Antioxidant Activity

Antioxidant activity of turmeric and black pepper was determined by *in vitro* antioxidant assay, i.e., 1,1-diphenyl-2-picrylhydrazyl (DPPH) free radical scavenging activity. The method developed by Blois was used with slight modification to access DPPH free radical scavenging activity [22]. In brief, 0.1 mM solution of DPPH was prepared in ethanol (99% purity) and 0.5 mL of this solution was added to 1.5 mL of curcumin solution in ethanol at different concentrations (15-45 *μ*L/mL). These solutions were vortexed thoroughly and incubated in the dark for 30 minutes. A half hour later, the absorbance was measured at 517 nm against blank samples, lacking the scavenger. Ethanol (99%) was taken as blank, while DPPH solution in ethanol (99% purity) was taken as control and butyl hydroxyl toluene (BHT) was used as a standard reference synthetic antioxidant with *R*^2^ value ranging from 0.95 to 0.99. The scavenging effect was calculated using the equation given ahead.

(4) $$\begin{array}{c} \text{DPPH scavenging effect }\left(\%\right)=\left(1-\frac{A_{\text{s}}}{A_{\text{c}}}\right)\times 100, \end{array}$$

where *A*_s_ is the absorbance of the sample and *A*_c_ is the absorbance of control samples.

### 2.8. Total Phenolic Content

The total phenolic content of gummies was measured by using a standard method [23]. One gram sample was weighed and ground in a pestle and mortar in 10 times the volume of 80% ethanol. The sample was centrifuged at 10,000 rpm for 20 minutes to homogenate. The residue was reextracted with 5 times the volume of 80% ethanol (99%) and centrifuged. The supernatant was evaporated to dryness, and 5 mL of distilled water was added to the residue. From the sample extracts, 0.2 mL was pipetted out in a test tube and volume was made up to 3 mL to which 0.5 mL Folin-Ciocalteu reagent was added. After 3 min, 2 mL of 20% Na_2_CO_3_ was added and thoroughly mixed; then, samples were placed in a boiling water bath for exactly 1 min. Samples were then cooled, and the absorbance was measured at 650 nm by using a spectrophotometer against a reagent blank. The standard curve was prepared using different concentrations of catechol. The total phenols in the sample were expressed as mg/100 g of the sample material.

### 2.9. Texture Profile Analysis

The texture of gummies was measured according to the modified method of Delgado and Bañón [17]. The universal stable texture analyzer TAXT2i (Stable Microsystems) was used for texture analysis. Probe SMSP/75 was used for measuring force on compression. Force calibration of the instrument was done prior to the start of the experiment to minimize measurement error. The instrument was operated at pretest speed = 10.00 mm/s, test speed = 0.5 mm/s, posttest speed = 10 mm/s, distance = 30 mm, strain = 65%, and trigger force = 5 g. The tests were performed at room temperature.

### 2.10. Sensory Evaluation

Hedonic scale is a method used in sensory evaluation to measure the degree of liking or acceptance of a product by individuals or a panel of judges. It typically ranges from 1 to 9, where 1 being extremely disliked to 9 being extremely liked. This scale provides a structured approach for participants to rate their perception of various attributes of the products, such as texture, appearance, flavour, and overall acceptability. Sensory evaluation of the developed gummies was carried out by a panel of semitrained judges who were asked to evaluate the products for different quality attributes, viz., texture, appearance, flavour, and overall acceptability, on 9-point hedonic scale [24, 25].

### 2.11. Statistical Analysis

All raw textural and sensory data was obtained and further analyzed using OPSTAT [26]. The data were subjected to analysis of variance using a factorial completely randomized design (CRD) at a 5% level of significance (*p* ≤ 0.05). The mean of different treatment samples was analyzed, and critical difference and standard error were used to compare and evaluate the significance of the sample values. Randomized block design (RBD) was used for sensory analysis experiments.

### 2.12. Formulations of Different Levels of Agar-Agar and Guar Gum Gummies

**Figure 1:**
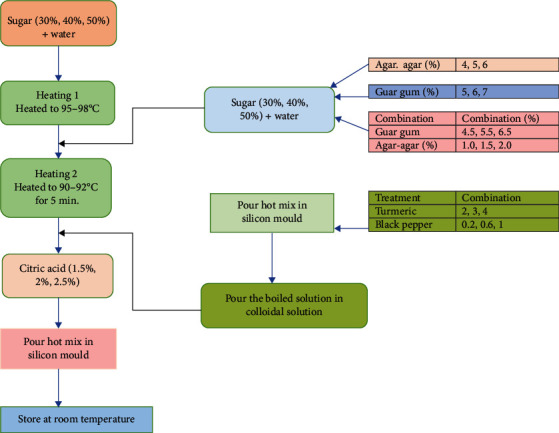
Process flowchart for vegan gummies supplemented with turmeric and black pepper.

In this experiment, the effect and properties of agar-agar and guar gum were evaluated with sugar and citric acid for the preparation of gummies. No artificial colouring and flavouring agents were added. The production of plant-based gummies involved a meticulously structured methodology to investigate the influence of sugar concentrations on the textural properties of the final product. The experimentation followed a systematic approach, beginning with the selection of suitable plant-based hydrocolloids, including agar-agar and guar gum, recognized for their gelling properties in food applications. To formulate the gummy base, a range of sugar concentrations (30%, 40%, and 50%) was meticulously chosen to explore the impact of varying sweetness levels on the final texture. A colloidal solution was prepared by dissolving agar-agar (4-6%) in water while continuously stirring until thorough mixing was achieved. Subsequently, citric acid (1.5–2.5%) was added to the solution. The total solution volume was standardized to 100 grams by carefully combining the recommended amounts of ingredients and adding water. The process commenced by dissolving the chosen plant-based hydrocolloids in water at specific temperatures to achieve optimal hydration and dispersion within the matrix. Subsequently, the predefined concentrations of sugar were gradually introduced into the hydrocolloid solution under controlled mixing conditions, ensuring homogeneity before gelation. The gelation process involved meticulous temperature control and timing to achieve the desired chewy texture analogous to traditional gelatin-based gummies. Each batch underwent a standardized cooling and setting phase to allow the hydrocolloid-sugar matrix to solidify into the gummy form (Figure 1). Notably, the textural attributes, including firmness, elasticity, and chewiness, were assessed using established instrumental methods (e.g., texture profile analysis) and sensory evaluations conducted by a trained panel. This methodological approach is aimed at elucidating the role of varying sugar concentrations in tandem with plant-based hydrocolloids, shedding light on their synergistic effect in mimicking the texture of gelatin-based gummies. Texture analysis was conducted using a texture analyzer to assess parameters such as chewiness, gumminess, springiness, adhesiveness, and firmness. Furthermore, each sample underwent evaluation by a semitrained sensory panel to assess various quality attributes, including texture, appearance, flavour, and overall acceptability, rated on a 9-point hedonic scale. Subsequently, formulations that received the highest scores in sensory evaluations were selected for further investigations and studies. The best formulation of plant-based hydrocolloids used in combination was selected based on chewiness and gumminess which was then used for the incorporation of turmeric and black pepper powder. Different concentrations of turmeric powder and black pepper powder were added to find the most suitable formulation based on sensory evaluation. Turmeric and black pepper-based gummies were prepared in which concentrations of turmeric powder (i.e., 1-3%) and black pepper powder (i.e., 0.2-1%) were added and prepared by the procedure mentioned in Figure 1.

## 3. Results and Discussion

### 3.1. Effect of Agar-Agar, Guar Gum, and a Combination of These Hydrocolloids on the Chewiness and Gumminess of Vegan Gummies

The texture is a primary determinant of quality in gelled confectionery products and, due to their relatively simple composition, provides an ideal model for mixed gel investigations [27]. Gumminess and chewiness are texture analysis descriptors that are particularly applicable to gelled confections [28]. The combination of hydrocolloids may affect the gelling properties of gummies which makes it necessary to modify procedure conditions to ensure proper gelation [29]. We examined the effects of the agar-agar and guar gum alone and in combination on the gelation network formation and textural properties through the texture profile analysis.

#### 3.1.1. Effect of Agar-Agar on Textural Properties of Vegan Gummies

**Figure 2:**
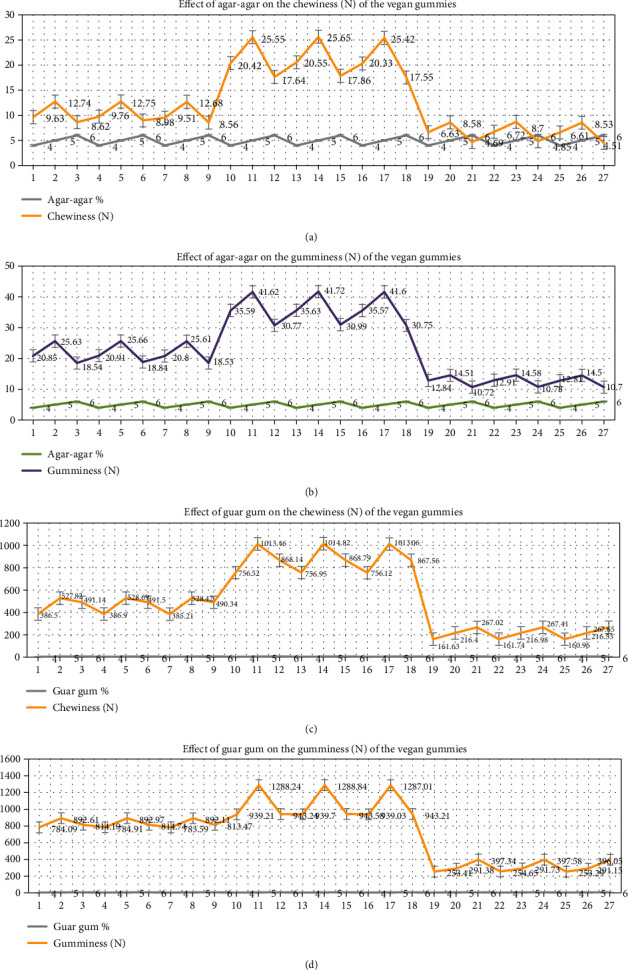
(a–d) Effect of agar-agar and guar gum on the chewiness (N) and gumminess (N) of the vegan gummies.

Different concentrations of agar-agar, sugar, and citric acid (5%, 40%, and 2%, respectively) were utilized in the creation of vegan gummies, and their chewiness (25.65 ± 0.81 N) and gumminess (41.72 ± 0.80 N) were evaluated using a textural profile analyzer. Agar-agar concentration statistically showed a significant difference at a 5% level of significance. With the increase in the concentration of agar-agar from 4% to 5%, the mean value of chewiness and gumminess was found to increase from 12.24 ± 1.36 N to 15.62 ± 0.77 N and 23.10 ± 0.68 N to 27.26 ± 0.74 N, respectively. However, a further increase in the concentration of agar-agar from 5% to 6% resulted in a decrease in mean value from 15.62 ± 1.24 N to 10.36 ± 1.12 N and 27.260.79 N to 20.06 ± 0.89 N, respectively (Figures 2(a) and 2(b)). It was observed that weak gel was formed when the concentration of agar-agar was less, i.e., 4%, whereas when the concentration of agar-agar was increased to 5%, proper gelation formation was observed along with maximum chewiness and gumminess. Furthermore, an increase in agar-agar concentration to 6% resulted in greater hardness due to the higher presence of galactan molecules participating in the gel matrix formation, leading to a less cohesive texture. This adversely affected the desired textural properties (chewiness and gumminess) of the gummies. As a result, in this application, 5% level concentration of agar-agar seems like the best concentration to pursue moving forward. A significant difference was observed at a 5% level for the combined effect of sugar, citric acid, and agar-agar on gummies' textural properties (chewiness and gumminess). Similar findings were observed in a previous study by Barrangou and coworkers in which they found an increasing trend between agar concentration in a gel and gel strength [29]. As the concentration of agar-agar increases in the gummy formulation, there are more galactan molecules present in the gel to participate in the formation of the gel matrix [30].

#### 3.1.2. Effect of Guar Gum on Textural Properties of Vegan Gummies

The concentration of guar gum had a notable impact on the textural characteristics—specifically, chewiness and gumminess—of the gummies at a 5% level of significance. In Figures 2(c) and 2(d), it is evident that lower concentrations of guar gum, such as 5%, resulted in reduced chewiness and gumminess, possibly indicating an imbalance in the ratio between water and guar gum. With the increasing concentration of guar gum from 5% to 6%, chewiness and gumminess increased proportionally probably due to the interaction of the galactose side chain of guar molecule with a water molecule in proper manner and it enhances the intermolecular chain interaction or entanglement which leads to increase in textural properties, i.e., chewiness and gumminess. The rise in concentration from 6% to 7% resulted in decreased chewiness and gumminess, likely due to a higher ratio of guar gum to water. This increased ratio led to heightened gel hardness, negatively impacting both chewiness and gumminess [31]. Guar gum gummies showed that chewiness and gumminess were 1014.82 ± 1.78 N and 1288.84 ± 1.56 N, respectively, when the concentration was 6%, 40%, and 2% for guar gum, sugar, and citric acid, respectively.

#### 3.1.3. Effect of Agar-Agar and Guar Gum Used in Different Combinations on Textural Properties of Vegan Gummies

**Figure 3:**
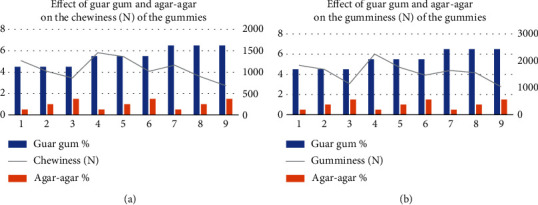
(a, b) Effect of different levels of guar gum and agar-agar in combination on the chewiness (N) and gumminess (N) of the vegan gummies.

**Figure 4:**
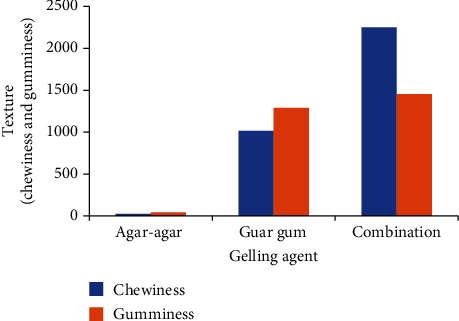
Effect of different plant-based gelling agents on texture profile (chewiness and gumminess) of gummies when used alone and in combination (agar-agar and guar gum).

The conducted study, illustrated in Figures 3(a) and 3(b), revealed the impact of varied concentrations of guar gum and agar-agar on the texture profiles of prepared gummies. Gumminess values ranged notably from 1417.70 ± 1.98 N to 1825.09 ± 2.67 N for guar gum and 1223.78 ± 2.74 N to 1909.42 ± 2.51 N for agar-agar. These distinctions proved statistically significant at a 5% level for both variables. The interaction between these variables showcased the highest gumminess (2251.11 ± 2.14 N) achieved at 5.5% guar gum and 1% agar-agar. While gumminess notably increased with rising guar gum levels from 4.5% to 5.5%, a subsequent decline occurred from 5.5% to 6.5%. This decrease was attributed not only to increased agar-agar concentrations from 1% to 2% but also to perceptions of decreased gumminess. Consequently, gummies formulated with 5.5% guar gum and 1% agar-agar demonstrated the highest gumminess. However, when using 0.5% agar-agar and 5.5% guar gum, chewiness and gumminess were recorded at 1455.12 ± 1.75 N and 2251.11 ± 2.14 N, respectively.  Figure 4 depicts the impact of various plant-based gelling agents—used individually and in combination (agar-agar and guar gum)—on the texture profile, specifically chewiness and gumminess, of vegan gummies. While gelatin-like textural properties were not observed when using agar-agar or guar gum alone, the combination of agar-agar and guar gum yielded a texture remarkably similar to gelatin-based gummies. This highlights the role of agar-agar in providing structural integrity to the gummy, complemented by guar gum, which contributed to its chewiness. It is noteworthy that hydrocolloids, such as these gelling agents, are commonly combined to impart distinct characteristics and textural attributes to gummies and jellies [32]. Research has shown that these plant-based gelling agents, when used in combination, can replicate some of the textural characteristics associated with gelatin-based gummies. Studies comparing the texture profiles of gelatin-based gummies to those of vegan gummies have indicated similarities and differences. For instance, while gelatin imparts a specific elasticity and chewiness to gummies, the combination of agar-agar and guar gum in vegan gummies has been found to provide a comparable chewiness and gumminess, contributing to a similar mouthfeel and texture [33].

### 3.2. Effect of Agar-Agar, Guar Gum, and a Combination of These Hydrocolloids on the Sensory Acceptability of Vegan Gummies

#### 3.2.1. Effect of Agar-Agar on Sensory Properties of Vegan Gummies

**Figure 5:**
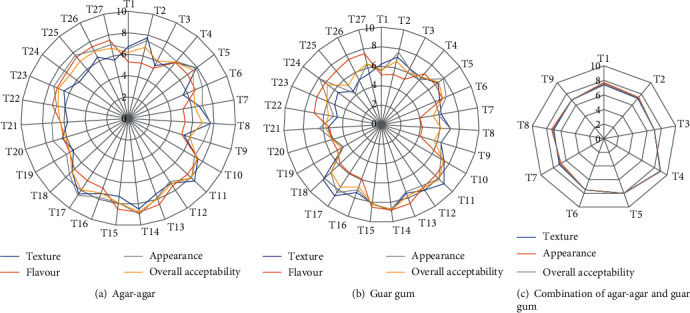
(a–c) Sensory scores of different levels of agar-agar and guar gum and a combination of both on vegan gummies.

Sensory evaluation plays a vital role in understanding how the structural and textural aspects of a product correlate with human perception. The study delved into the sensory data encompassing textural scores, flavour, appearance, and overall acceptability of gummies. These scores ranged notably from 5.4 to 8.5 for texture, 5.25 to 8.8 for flavour, 6.5 to 8.98 for appearance, and 6.0 to 8.76 for overall acceptability, as illustrated in Figure 5(a).

The sensory analysis revealed intriguing insights into how varying concentrations of sugar, citric acid, and agar-agar affected the perceived qualities of the gummies. For instance, according to the sensory evaluation results, formulations with 40% sugar, 2% citric acid, and 5% agar-agar garnered the most favourable responses across the board in terms of texture, flavour, appearance, and overall acceptability. These sensory scores indicate that this specific combination offered a more appealing texture, desirable flavour, visually pleasing appearance, and higher overall likability among the panelists participating in the sensory evaluation. The link between agar-agar concentrations and sensory perceptions is crucial for product optimization. Sensory evaluations involving agar-agar concentrations in vegan gummies revealed that higher agar-agar levels tend to alter the texture and overall acceptability. The current study suggests that while agar-agar contributes to the structural integrity of gummies, an optimal concentration, as determined by sensory analysis, plays a pivotal role in achieving the desired textural attributes and overall sensory experience. Conclusively, the sensory analysis conducted in this study emphasized the significance of balancing agar-agar concentration alongside sugar and citric acid for the formulation of appealing vegan gummies. It showcased that the 40% sugar, 2% citric acid, and 5% agar-agar combination resulted in optimal sensory attributes, serving as a valuable guideline for gummy formulation. Knowledge of structure and texture is most useful when it can be related to human sensory opinion. In the study, sensory data indicate the effect of sugar, citric acid, and agar-agar on the textural score, flavour, appearance, and overall acceptability of gummies, which ranged from 5.4 to 8.5, 5.25 to 8.8, 6.5 to 8.98, and 6.0 to 8.76, respectively (Figure 5(a)). Based on sensory analysis, 40% sugar, 2% citric acid, and 5% agar-agar were optimized to make gummy formulation.

#### 3.2.2. Effect of Guar Gum on Sensory Properties of Vegan Gummies

To make gummies from guar gum gelling agent, 40% sugar, 2% citric acid, and 6% guar gum formulations were optimized. The study extensively evaluated the sensory impact of varying concentrations of sugar, citric acid, and guar gum on the perceived textural scores, flavour, appearance, and overall acceptability of the gummies. The sensory data, as illustrated in Figure 5(b), showcased a range of scores, from 4.51 to 8.86 for texture, 5.11 to 8.80 for flavour, 4.16 to 8.70 for appearance, and 5.22 to 8.78 for overall acceptability. The decision-making process behind the formulation optimization was multifaceted. It involved a meticulous analysis of how different concentrations of guar gum, in combination with sugar and citric acid, influenced the sensory attributes of the gummies.

#### 3.2.3. Effect of Agar-Agar and Guar Gum Used in Different Combinations on Sensory Properties of Vegan Gummies

The combination of hydrocolloids may affect the gelling properties of gummies which makes it necessary to modify process conditions to ensure proper gelation. The effects of the combination of agar-agar and guar gum on the formation of the gelation network were examined through sensory evaluation (texture, appearance, and overall acceptability). The total weight percentage of hydrocolloids in combination gummy formulation was kept variable, and other ingredients (sugar, citric acid) were kept constant. A synergistic effect on texture (chewiness and gumminess) was observed in the combination of agar-agar and guar gum, where agar-agar contributed to the proper structure of the gummy and guar gum enhanced textural properties like chewiness and gumminess. However, when used alone, agar-agar failed to achieve textural properties akin to gelatin, and similarly, when used independently, guar gum could not provide adequate structure to the gummies. The combination of 1% agar-agar and 5.5% guar gum proved effective in achieving desired textural properties (Figure 5(c)). The highest gumminess score (2251.11 ± 2.14 N) was observed at 5.5% guar gum and 1% agar-agar, indicating increased gumminess until 5.5% guar gum, followed by a decline at 6.5%. Combining 5.5% guar gum and 1% agar-agar resulted in the highest gumminess, while 0.5% agar-agar and 5.5% guar gum exhibited 1455.12 ± 1.75 N chewiness and 2251.11 ± 2.14 N gumminess. The combined effect of agar-agar and guar gum showcased a synergistic impact on texture (chewiness and gumminess). Agar-agar contributed to the gummy's structural integrity, while guar gum enhanced textural properties. However, when used alone, agar-agar could not replicate gelatin-like textural properties, and likewise, guar gum alone could not provide adequate structure to the gummies. Sensory data on the effective combination of 1% agar-agar and 5.5% guar gum for textural properties (Figure 5(c)) should be compared with texture profile analysis data to ascertain their mutual support.

### 3.3. Supplementation of Turmeric and Black Pepper Powder in Standardized Plant-Based Hydrocolloids of Vegan Gummies

**Figure 6:**
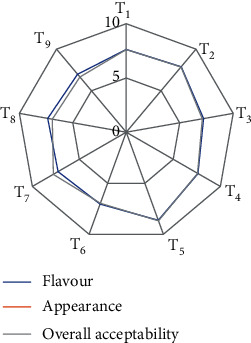
Sensory scores of different formulations of turmeric and black pepper powder-supplemented gummies.

From previous sections, the best formulation of plant-based hydrocolloids used in combination was selected based on chewiness and gumminess which was then used for the incorporation of turmeric and black pepper powder. Different concentrations of turmeric powder and black pepper powder were added to find the most suitable formulation based on sensory evaluation. Turmeric and black pepper-based gummies were prepared in which concentrations of turmeric powder (i.e., 1-3%) and black pepper powder (i.e., 0.2-1%) were added and prepared by the procedure mentioned in Figure 1. Different combinations of turmeric powder and black pepper powder-supplemented gummies were evaluated organoleptically, and the formulation with the highest score was taken for final optimized levels for vegan gummies. In the study, turmeric and black pepper were used to examine the effect on flavour, appearance, and overall acceptability and were found to show a significant difference at a 5% level statistically. The sensory score of flavour varied from 6.25 to 8.75 (Figure 6). As the concentration of turmeric and black pepper increases, the score for flavour increases till the concentration reaches 2%. After that, the sensory score of flavour decreased, as the concentration of turmeric and black pepper increased. The increasing bitterness of turmeric and pungency of black pepper might be the reasons behind this. The increasing bitterness of turmeric and pungency of black pepper can be attributed to several chemical compounds present in these spices. Turmeric contains a compound called curcumin, which contributes to its vibrant colour but can also impart a bitter taste, particularly when used in higher concentrations. As the concentration of curcumin increases, so does the bitterness. Black pepper's pungency is mainly due to a compound called piperine. Piperine is responsible for the spicy, pungent taste of black pepper. When present in higher amounts, it intensifies the spiciness and pungency of the spice. Research has shown that the bitterness of turmeric is directly linked to the concentration of curcuminoids, particularly curcumin, demethoxycurcumin, and bisdemethoxycurcumin [34]. Similarly, the pungency of black pepper is associated with the amount of piperine present, with higher concentrations leading to increased spiciness [35]. The interactions between the compounds in these spices can influence the overall flavour profile when used together. According to literature, it was found that piperine from black pepper increases the bioavailability of curcumin 2000%, when added 2 mg of piperine with 2 g of curcumin [14].

The highest mean score for appearance was observed as 8.625. The overall acceptability score was higher (8.7). The higher overall acceptability of the gummies might be attributed to the inclusion of plant-based gelling agents like guar gum and agar-agar, combined with turmeric and black pepper. Based on the overall acceptability score, specific levels of turmeric and black pepper were determined, and these findings were used to create the standardized formulation for vegan gummies enriched with turmeric and black pepper in Table 1.

### 3.4. Physicochemical Analysis of Standardized Vegan Gummies Supplemented with Turmeric and Black Pepper

The comparison of the physical parameters and chemical composition of the prepared turmeric and black pepper vegan gummies with existing literature (Table 2) highlights the importance of aligning within certain ranges established by previous studies. The moisture content of the turmeric and black pepper vegan gummies, at 11.21 ± 0.46%, falls within the range observed in other gummies documented in the literature [18]. This alignment is crucial as moisture content influences the gummies' texture, shelf stability, and microbial safety. Similarly, the water activity of the standardized gummies, recorded at 0.71 ± 0.028, reflects a range consistent with previous studies [36, 37]. This parameter is pivotal for predicting microbial growth and ensuring product stability during storage, emphasizing the significance of maintaining this range within established limits. However, certain deviations were noted in specific chemical constituents. Although the pH value and total titratable acid of the gummies fell within the ranges reported in earlier gummy studies [38, 39], the total soluble solids (TSS) value slightly deviated, being marginally lower. Additionally, the curcumin content was observed to be slightly higher compared to similar gummies documented in the literature [18]. These variations indicate potential differences in flavour intensity, colour, and possibly nutritional composition. In the case of black pepper vegan gummies, the total phenolic content, antioxidant capacity, and piperine were measured at 56.9 ± 0.09 mg/100 g, 37.27 ± 1.4%, and 0.02 ± 0.008%, respectively. These findings offer insights into the gummies' nutritional value and potential health benefits, aligning with or differing slightly from reported values in prior studies. Overall, while many attributes of the prepared gummies fall within the expected ranges from previous literature, slight variations in certain chemical constituents like TSS, curcumin content, and phenolic levels highlight nuances in taste, colour, and potential health-promoting compounds that may distinguish these gummies from those previously documented. Turmeric is renowned for its active compound curcumin and celebrated for its potent antioxidant and anti-inflammatory properties. Its higher-than-average presence in the prepared gummies, although not directly compared to existing gummy formulations, suggests a potential boost in the product's bioactive properties. Curcumin's health benefits, including its role in reducing inflammation and combating oxidative stress, are well documented in scientific literature [40]. Black pepper, on the other hand, contains piperine, which is recognized for enhancing nutrient absorption and exhibiting antioxidant effects. Its presence in the gummies highlights the potential for improved nutrient uptake, contributing to overall health benefits. The absence of literature on the specific combination of turmeric and black pepper in gummies underscores the novelty of this formulation. By integrating these two potent herbal ingredients into vegan gummies, this novel approach capitalizes on their synergistic effects. Turmeric's curcumin, when combined with black pepper's piperine, may potentially enhance bioavailability and maximize the health benefits of both compounds [41]. This unique combination holds promise in offering consumers a convenient and palatable way to access the holistic health advantages associated with these herbs. Moreover, the physical texture parameters like hardness, chewiness, gumminess, and springiness are crucial indicators not only for sensory satisfaction but also for the release and absorption of bioactive compounds in the body. In essence, the incorporation of turmeric and black pepper into vegan gummies marks a pioneering step in utilizing these renowned herbs for health enhancement. Despite lacking direct comparisons, understanding the health implications of their constituents emphasizes the potential value and uniqueness of this innovative herbal combination.

## 4. Conclusion

In conclusion, this study demonstrates the successful development of vegan gummies supplemented with turmeric and black pepper, utilizing a combination of plant-based gelling agents, namely, 1% agar-agar and 5.5% guar gum. The optimized formulation achieved desirable textural properties, including a chewiness of 1,455.12 ± 1.75 and gumminess of 2,251.11 ± 2.14. With sugar at 40% and citric acid at 2%, the gummies exhibited sensory acceptability. Furthermore, the incorporation of turmeric and black pepper powder was optimized at concentrations of 2 g and 0.6 g, respectively. The cost-effectiveness of these “anytime-everywhere” vegan gummies is comparable to commercial alternatives. Future research may explore additional plant-based gelling agents and address packaging, storage, and commercialization for enhanced convenience. By delving into these areas, the study can expand its scope towards practical application, market viability, and consumer acceptance of the “anytime-everywhere” vegan gummies supplemented with turmeric and black pepper. This holistic approach will pave the way for potential commercialization and market success. For future work, targeted studies could focus on investigating novel plant-based gelling agents beyond those explored in this study. Additionally, in-depth investigations into packaging materials and storage conditions could optimize shelf-life and product stability. Collaborative efforts with packaging experts and market research specialists may provide invaluable insights for successful commercialization strategies. These combined efforts will fortify the path towards effective commercialization and market penetration of the innovative turmeric and black pepper-infused vegan gummy product.

## Figures and Tables

**Table 1:** Standardized formulation for optimized levels of turmeric and black pepper powder in vegan gummies.

Ingredients	Quantity
Sugar	40 g
Citric acid	2 g
Guar gum	5.5 g
Agar-agar	1 g
Turmeric	2 g
Black pepper	0.6 g
Water	48.9 g

**Table 2:** Physicochemical analysis of standardized vegan gummies supplemented with turmeric and black pepper.

Characteristics	Mean ± SD	Literature values	References
Moisture (%)	11.21 ± 0.46	10.47-13.23	[18]
Water activity	0.71 ± 0.028	0.5-0.75	[37]
Total soluble solids (°Brix)	68.5 ± 1.2	70.99-78.31	[38]
pH	3.5 ± 0.24	3.0-5.0	[39]
Antioxidant capacity (%) (DPPH)	37.27 ± 1.4	—	—
Total phenols (mg/100 g)	56.9 ± 0.09	—	—
Total titratable acid (g/100 g)	2.88 ± 0.56	2.78-3.59	[42]
Curcumin (%)	0.054 ± 0.0012	0.03	[18]
Piperine (%)	0.02 ± 0.008	—	—
Hardness (g)	4023.16 ± 15.38	1500-12,000	[43]
Chewiness (N)	1332.37 ± 9.43	701-12,226	[42]
Gumminess (N)	2105.013 ± 15.45	500-7617.6	[43]
Springiness (mm)	0.62 ± 0.54	0.96-1.00	[42]

## Data Availability

The data given in this study are available in Krishikosh, an institutional repository of Indian Agricultural Research System, at https://krishikosh.egranth.ac.in/ without any reference number.
